# Living with Uncertainty: Older Persons' Lived Experience of Making Independent Decisions over Time

**DOI:** 10.1155/2013/403717

**Published:** 2013-03-07

**Authors:** Agneta Breitholtz, Ingrid Snellman, Ingegerd Fagerberg

**Affiliations:** ^1^Department of Neurobiology, Care Sciences and Society (NVS), Karolinska Institutet, 141 83 Huddinge, Sweden; ^2^School of Health, Care and Social Welfare, Mälardalen University, P.O. Box 883, 721 23 Västerås, Sweden; ^3^School of Health, Care and Social Welfare, Mälardalen University, P.O. Box 325, 631 05 Eskilstuna, Sweden; ^4^Department of Health Care Sciences, Ersta Sköndal University College, P.O. Box 11189, 100 61 Stockholm, Sweden

## Abstract

The aim of the study was to illuminate the meaning of older persons' independent decision making concerning their daily care. Autonomy when in care is highly valued in the western world. However, research shows that autonomy can give rise to problematic issues. The complexity of independence and dependence for older people when living at home with help has also been highlighted. In Sweden, older people are increasingly expected to live at home with help from municipal home care services, and study into this aspect of care is limited. This study is a part of an ongoing project and has a qualitative life world perspective. Audiotaped narrative interviews were conducted and analysed using a phenomenological hermeneutic method. Findings revealed a main theme: “living with uncertainty as to how to relate one's own independence and dependence with regard to oneself, and others.” This involves a constant process of relating to one's independence controlled by others or oneself, and adjusting one's independence and dependence with regard to oneself and others. The conclusion is that professional carers need to acknowledge the changing vulnerability of dependent older persons over time. The implication is a relational approach to autonomy beyond the traditional individualistic approach.

## 1. Introduction

The care of the old, with an increasing population over 60 years old, presents a challenge worldwide [1]. In Sweden municipalities are responsible for the care of the old, and older people are now increasingly expected to live at home aided by municipal home help services. As a consequence older people's care needs have increased as well as the workload for professional carers [2]. Older people apply for support, and the help available includes laundry, cleaning, shopping, personal care, meals, and emergency alarms. Care managers assess their needs according to the Social Service Act, which requires that people's right to self-determination and integrity should be respected [3].

Autonomy when in care is highly valued in the western world and involves people's right to make their own choices without involving others [4–6]. Nevertheless, in the care of the old this individualistic approach to autonomy is problematic due to their dependence on others in their everyday lives [4, 7–9]. However, Sandman [6] argues that it is important to recognize that people value their autonomy differently. Further, it is therefore important to distinguish between different aspects of autonomy, that is, self-determination, freedom, desire fulfilment, and independence. The most central aspect is considered self-determination and means how people make decisions in accordance with their own will. Whereas independence means how people perform or carry out decisions regardless if they decide to do it themselves or hand it over to someone else to decide. Olaison and Cedersund [10] found that in assessment meetings between care managers and older people applying for home care the focus was on fixed standard solutions based on social service practice, and older people had to negotiate within the standard solution context to make clear their individual needs. Research into older people in need of home help and care highlights the complexity of independence and dependence. Independence for older people has both positive aspects, for example to be able to make one's own decisions, and negative aspects in terms of isolation and underestimating one's needs [11]. In a study [12] with older people of their experiences of frailty and followed over time, findings show the complexity of how they balanced between autonomy and dependence. The findings of Hammarström and Torres [13] show the complexity of older people's striving for self-determination at the same time as accepting their dependency. However, Welford et al. [14] reveals in a concept analysis of autonomy for older people in residential care that professional carers can improve older people's autonomy in caring relationships and create the opportunity for them to be involved in decision making based on their own abilities. 

Studies into older people's independent decision making using municipal home help services appear to be rare. Nevertheless research shows the complexity of the balance between independence and dependence for older people in home help care. In addition the findings in Breitholtz et al. [15] study reveal that the older persons struggled for the opportunity versus resigning themselves to losing the opportunity to make their own decisions. This was further understood as the older persons are not being treated as individuals, not having their needs met, and their life situation being stressful. These findings were considered important and inspired us to further deepen the understanding of the older persons' lived experiences of making independent decisions. This study is a part of an on-going project adopting a qualitative and life world perspective [16] to illuminate the meaning of older person's independent decision making and of their professional carers to enable this. Accordingly, the focus is on the meaning of the lived experiences. The aim of this study was to illuminate the meaning of older persons' independent decision making concerning their daily care. 

## 2. Materials and Methods

### 2.1. Participants

The participants were seven older persons who had been part of the project from the start and who are presented elsewhere [15]. The inclusion criteria were aged 70 or more, both men and women, being able to speak and understand Swedish, and varied levels of care needs and still living alone at home with daily help from municipal home help services. In addition they were cognitively screened prior to enrolment and before data collection for this present study according to MMSE [17], requiring a score of at least 24 out of 30. Before data collection commenced staff managers phoned the old people to ask if they were still interested in participating and being cognitively screened again. After they had been screened and given their verbal consent to continue participating they were phoned by the first author and an appointment for a second interview was set up. Participants were between 80 and 91 years old, with four from one municipality and three from another, and with one man and six women. They still lived alone at home with professional carers visiting between one to six times a day. The domestic help provided consisted of tasks such as preparing meals, cleaning, washing up and making up beds, and care help with such matters as personal hygiene.

### 2.2. Data Collection

In order to deepen the meaning and understanding of the participating older persons' lived experiences of independent decision making, they were each interviewed three times in October and November 2009 and January 2010. The interviews were conducted during and after one of the participating older persons' professional carer, they were paired with, participated in an educational programme. The same [15] open-ended interview guide was used with open questions such as the following. Can you tell me what it is like when professional carers help you with your daily care? Can you tell me how you experience the opportunity to make your own decisions about your daily care when a professional carer is helping you? To deepen the understanding follow-up questions were asked such as the following. Can you tell me more about what you thought about that? Can you tell me some more about how you felt? Participants were encouraged to narrate as freely as possible about their lived experiences of making independent decisions. The interviews were conducted in the older person's homes and lasted between 40 to 65 minutes.

### 2.3. Data Analysis

Data analysis followed the phenomenological hermeneutic methodological stages: naïve reading, structural analysis, and comprehensive understanding with a dialectic movement between explanation and understanding [18, 19]. The three interviews with each participant were first separately transcribed and read and a first naive understanding was formulated for each of them. Thereafter, all interviews were analysed as a whole to enable an in-depth understanding of the older persons lived experiences. A naïve understanding was formulated and guided the succeeding structural analysis. This analysis began with dividing the whole text into meaning units expressing one meaning and those were further condensed to formulate the essential meaning as briefly as possible. The condensed meaning units were then abstracted in an on-going process into sub-themes, themes and a main theme (see Table 1). They were further reflected upon with the naive understanding in mind, in an on-going process to validate the naïve understanding. A comprehensive understanding was formulated through reflections on the interview texts, naive understanding, main theme, themes and sub themes, the research question, authors' preunderstanding, and relevant literature to deepen the understanding [18].

### 2.4. Ethical Considerations

This study was approved by the Regional Ethics Committee (ref. 2008/256). Participants were informed in advance [15] that they would be paired with a professional carer, followed over time and cognitively screened again prior to their continued participation. Confidentiality was assured and an informed consent was signed and collected before the first interview. They were contacted by the staff manager before data collection for this study and asked if they still wanted to participate. This procedure was chosen primarily to obtain a declaration that the older persons receiving municipal home help service still wanted to participate and that their health had not declined since the first interview was conducted in spring 2009. 

## 3. Findings

### 3.1. Naïve Understanding

Making independent decisions is conditional on the other people involved in the organization. One's life is a part of that organization that includes all the people being involved, and one tries to understand how one fits in. To make it smoother for all persons involved, one complies and sets aside one's own needs and wishes. One's own ability to express needs and wishes are limited by the fact that the decisions and regulations within the organization are outside one's own control and that of professional carers. To expect the arrival of a particular professional carer is both joyful when they show up but also disappointing if they do not. The endless wait for them to come limits one's own freedom or induces feelings of fear of being left alone and not being able to take care of oneself in one's own home. Deciding for oneself is to have the opportunity to participate in one's own care and a trustful relationship with professional carers, giving and taking with each other. The wish is to be independent and professional carers are an extended arm no matter who provides the help. Yet one's own dependency changes over time and it feels safe to hand over responsibility to a professional carer to decide about one's daily care. It may involve an implicit struggle and a wish that the responsibility should rest in their hands, but it is not taken for granted that they acknowledge one's spoken or unspoken needs. The ability to receive help from one's surroundings makes one feel less dependent on one's professional carers. Not wanting to be a bother to one's relatives makes oneself feel more dependent on professional carers.

### 3.2. Structural Analysis

The findings from the structural analysis are presented in a main theme following themes and their subthemes (see Table 2).

#### 3.2.1. Living with Uncertainty as to How to Relate One's Own Independence and Dependence with regard to Oneself and Others

Making independent decisions in daily care over a period of time involves “Living with uncertainty as to how to relate one's own independence and dependence with regard to oneself and others.” Being aware of one's own vulnerability to dependence on others and still wanting to be independent presents a life-changing situation, as one tries to comprehend one's everyday life. This comprehension involves relating to one's own independence and dependence on others, but this raises uncertainty because one's dependence changes over time and is affected by circumstances within the organizations and professional carers' working conditions. It makes it complicated and one has to live with uncertainty every day since one never knows who is going to come. Although one has a particular professional carer who provides special attention over a period of time, there is still no guarantee that one's needs and wishes will be fulfilled, and one is still uncertain when this person will come. Being uncertain makes one dependent on others such as relatives and other people in the organization, and one tries to comprehend how to make it work for all persons involved.

#### 3.2.2. One's Independence Lies in the Hands of Others

When* relying on others to manage one's life *one has to strive for independence with the help of others. Feeling independent depends on what help one may get from professional carers and close friends and family. On the one hand being able to get help from relatives, friends, or the private sector facilitates a decreased independence on one's professional carers. On the other hand, one's desire to not burden relatives induces an increased dependence on professional carers.

*Well, I just told her that we only can have one lamp on in the bathroom. Directly she says, but then I can help you I can fix that for you she said. Well at that time I had already had my breakfast so she didn't have to make any breakfast for me. Afterwards when I had taken the shower and my foot was fixed, well she reckoned she had the time to fix it for me. But otherwise I never ask for help like that, because I have my children who help me with many things. I usually try to let them fix things like this for me. But otherwise it is no problem for me to ask for help with things and I get it done.*

Independence is relying on the willingness of others to help, and this is something one tries to handle to organize life in the best interest of all persons involved.

*Deciding for oneself is beyond one's reach* when others are making decisions about one's needs and without having the opportunity to express one's wishes due to the time-pressured working of professional carers. One has no opportunity to arrange their work schedules, and one therefore, has to accept that one may have to meet different people every time. One's own needs and wishes are not fulfilled when one has to follow their work schedules without the opportunity to express what would be appropriate for oneself.

*Well they just bring what I want, but I do not like though that they are not allowed to heat in a pot nor in a frying pan. They are only allowed to heat food up in the microwave. It becomes quite monotonous. They are not allowed to fry eggs or anything like that. There is a lot that you would like to have that can only be fried, but they are not allowed to do that. Well, it is just that you have to have stuff that goes in the microwave and it's not good, an omelette would be nice. No, they are not allowed under any circumstances. Well, it would feel like a relief because I could shop differently than I do now.*

Care managers assess one's needs and professional carers have to follow their decisions with no opportunity for oneself or them to change these. Having to ask care managers for support on every single help need complicates everyday life. 

*Waiting for others to come *not knowing at what time professional carers will arrive limits one's freedom to live in accordance with one's life plans. It prevents one from making plans for everyday life, when you are sitting and waiting for them. Even though one can leave home it is not good knowing someone is there while one is out. Not knowing when they show up can also induce a feeling of limited freedom and of being disturbed trying to live one's life, but also a fear of being abandoned and not being able to take care of oneself.

*… It is tough very very tough, well phew…. That is really the worst part of it, being in the bed well, because then I am vulnerable. That is why I tell them every time when they leave, make sure the door is locked. Because it would be awful if someone broke in, well there would be absolutely nothing you could do… ugh….*

Expecting and waiting for a particular carer that one trusts is a joyful experience, but disappointing if they do not turn up. One just waits and gets ready for someone in particular to come who can fulfil one's wishes and needs. 

#### 3.2.3. One's Independence Lies in One's Own Hands

*Deciding for oneself when to become involved* and having the opportunity to decide, when expressing one's wishes for encouraging and responsive professional carers, is good. It is also good having control over situations, knowing the best way to carry out chores for one's own personal well-being, and making one's own decisions in line with one's wishes when one has different alternatives to choose from. Not being restricted in one's own private home allows one to be free to decide.

**…* I do not want anything more on the table than this, one newspaper, perhaps a crossword and then the medicine that is what I want, and those pens as well. They do not care a bit, and say that my table is a mess, they never do care. But, I just tell them, I want this stuff, that's it. But no one has ever said something like that, like someone has said do not have it this way, put it this way or that way instead. No they couldn't do that….*

Arriving at the best solutions with professional carers allows one to decide. When they have time to listen to one's wishes it enables one to decide for oneself in cooperation with them, like being asked if there is something more one needs help with or feeling free to ask for more help. 

When* managing oneself with professional carers as an extended arm*, although being dependent, the help given infuses a feeling of independence. It does not matter which one of them turns up since they are all equal. One can take care of oneself, just having them as an extended arm performing chores one is not capable of alone.

*… I do not see any difference between the girls, they do exactly what I tell them. They just stand next to me watching when I step into the shower, all of them do. Then when I sit there washing, which I want to do myself, they leave and make up the bed. Then I just call out and tell them to help me wash my back, which they do. Then they leave and do whatever they have to do, like washing the dishes. I do not know what they are doing since it is only washing the dishes and making the bed they are supposed to do in the morning. Well, it feels good, I just call out and tell them to come.*

Receiving help is just like doing it oneself, seeing professional carers as an extended arm. It is oneself who has control over the situation and one either tells them what to do or they already know. Sometimes nothing needs to be said, perhaps just a brief chat, and just get help with daily chores, and then they can leave.

*Handing over the decisions to others* when one's independence changes over time feels reassuring, although the wish is to be as independent as possible. To be as independent as possible yet dependent in any given situation when one has a professional carer standing by as support is the ideal. It is easier to be offered help by those who take the initiative. When the body fails to perform as it should it feels safe to hand over the responsibility to a reliable professional carer who knows exactly where one's belongings are.

*Well, it was like this morning when she went into the closet in the hallway and found my pillowcases, which the others couldn't find because I did not know where I had put them. Consequently they were put on a shelf a little bit higher up were they couldn't find them. Now it turned out that the one who had taken care of the laundry and put it away had put it in another place. But she picked it out. Well it is my home, but I do not look after it all on my own, since we are so happy together so she can do whatever she wants here. She knows how to put the clothes away, and she does it without me asking her. If I want a particular pair of trousers she knows in which closet to look and she picks them out for me.*

One needs to be aware of one's varying vulnerability in life, and one's ability to take care of oneself differs from time to time. In a changing life situation, it feels reassuring to be cared for by a professional carer one can trust, to remind oneself of one's value as a human being.

*Giving and taking in the relationship with professional carers* when one has a mutual relationship with them increases the opportunities to decide for oneself. It feels good to have contact with those one can discuss one's personal interests with. To get help from those who encourage one to manage things infuses confidence and one feels more independent. A mutual and trusting relationship takes time to develop and one needs to be devoted.

*… Of course she has learned because she has been here so many times with me in different situations. We do not only talk about how she helps me but we have private chats as well. How life goes for her and how life has been for me, so there is a very good communication between us. Well it seems quite natural together with her….*

To give and take in the relationship is comforting and to feel respect for one another shows a willingness to make it easier for each other. Being persuaded for one's own good by a professional carer one trusts makes it easier rather than more difficult to fulfil one's wishes.

#### 3.2.4. Adjusting One's Own Independence and Dependence with regard to Oneself and Others

Neglecting one's own need to allow for the needs of others when one sees and hears professional carers in a hurry and knowing that there are other caretakers in more need of their attention and care is also a situation to consider. One must also realise what they are authorized to do and allow for this when trying to persuade oneself to manage, as long as one gets help with the most urgent chores.

*No, but I see by looking at them that they are busy. No, but then I think it may be why I do not ask for help. No, I don't because if I need help with something it may not be so urgent. If it is, I ask them and then they help me. But otherwise you can just see by looking at the different way they react.*

When feeling a sense of loyalty towards professional carers and other caretakers one's own needs are not so important compared to theirs. When seeing that they are stressed one feels that one does not want to bother them and accept the fact that there is no time. Although some small talk with them would be appreciated one still has no expectations and in some instances they are not even allowed to sit down and talk.

*Withholding one's thoughts* how the professional carers perform their task implies a struggle taking place. One wants professional carers to understand, but even though one's own needs and wishes are not fulfilled one still says nothing. If they do things without asking it may be difficult to sort it out afterwards. It is hard correcting their mistakes while it is easier just to let it go even though one is not happy about it.

*It is bad, when for example, they have done the laundry and hung up the clothes which have been in the washing machine; they never ask where to put it, they just put it anywhere. Then I do not know where it is. I do not know if it is me being stupid, but I do not want to push them too much either. Well, I think that I can correct it later. *

One observes the professional carers and their different ways of working while some of them take their time others just rush around and then leave. One goes along with it and has thoughts of being nice and gentle and hoping to get one's own needs and wishes fulfilled. One tries to adjust to their personalities and adopt what one thinks is an appropriate approach. This is something one gradually finds strategies for.

## 4. Comprehensive Understanding and Reflections

The findings with the same group of older persons [15] revealed that they struggled for the opportunity versus resigning themselves to losing the opportunity to make their own decisions. In this present study, the findings reveal a changed understanding of older persons' independent decision making as a life situation involving living with uncertainty over time. Older persons are aware of their own vulnerability and dependence but still want to be independent. They try to comprehend everyday life and this involves a movement, a dynamic process over time changing from day to day which makes them more vulnerable. Ricœur et al. [20] states that the starting point for reflection is via objectives and opens up the world for humans to acknowledge their needs and desires and what is lacking. It opens up as a sign to offer for others to recognize in mutuality even in the isolation of suffering (pages 18-19). In the present study the findings reveal that older persons adjusting own independence and dependence with regard to oneself and others, implying that there may be a struggle in wanting professional carers to understand their needs. This is further understood as an “implicit struggle” means that the old open up and invite professional carers to enter into a mutual understanding, wanting them to recognize their exposed situation along with their suffering. Accordingly when the professional carers fail to acknowledge this invitation and their needs it makes the older persons vulnerable.

As patients become more dependent increased attention is needed to enhance their well-being [21]. Autonomy is highly valued in the western care context [4–6]. Sandman [6] suggests that it is important to distinguish between four different aspects of autonomy as they can be valued differently by people, and there is no easy norm to follow. The central aspect, *self-determination, *means how people decide and act in accordance with their own will and thus make decisions. *Freedom *is an aspect of having different valuable alternatives to choose from, while *desire fulfilment* is an aspect of actual outcomes of decisions. *Independence* is an aspect of involvement and accomplishment to do things themselves. When the old have to relate to when their independence lies the in hands of others they have reduced self-determination, freedom, desire fulfilment and independence due to internal and external organizational circumstances. On the other hand, when independence lies in their own hands they have the opportunity to be involved and to have freedom, desire fulfilment, and independence. Older persons' dependency changes over time like circumstances in organizations and professional carers working conditions. This complicates everyday life and they have to live with uncertainty since they never know how things will be. Research shows that the ageing process makes things one is surrounded by in the home be seen in a different way and that routines provide continuity for older persons to live independently [22]. Accordingly attention needs to be paid to how older persons perceive everyday life and the importance of continuity for them. In a study, findings show that frail older people living at home with help experienced little support for their efforts in their everyday life [23]. Agich [4] argues that dependent older people have to accept being placed in the hands of others, relying on others to recognize their needs and help them. In this study, it was found that when older persons' independence lies in the hands of others it was interpreted as professional carers not recognizing their everyday life, which results in a routinized care. They also adjust to their independence and dependence, are aware of their dependency, and accept the situation. This is in line with the findings of Anderberg and Berglund [24] who found that when communication failed between the old and professional carers in nursing homes the old hid their vulnerability in order to be accepted. Ricœur et al. [20] point out that it is human limitations that make man fallible and fragility provides the opportunity for evil to arise (page 146). When autonomy is so highly valued in today's society it is easy to say that dependency is negative. Instead perhaps, the focus should change to help vulnerable older people and see their dependence as something human. Professional carers should acknowledge older peoples' changing vulnerability in everyday care and not just focus on independence and respect for self-determination. However, findings reveal that mutual caring relationships increase the opportunities for older persons to decide in accordance with their own needs, to be free to decide whether or not they want to hand over to professional carers to decide for them, or to decide for themselves or together with their carers. Nevertheless they are still uncertain because they never know when they will arrive. Dependence becomes accepted if patients feel free to be dependent in a mutual understanding with nurses [25]. This underlines a care with a person-centered perspective [26] and a change from an individualistic approach on autonomy towards a relational to focus on interdependence [4, 9, 27] to enable shared-decision making [28]. This relational perspective could be useful for professional carers to help vulnerable older people in caring encounters [29] to make their own decisions [6].

## 5. Methodological Considerations

In a life world perspective, it is important to have varied characteristics and experience amongst the participants [16] which was achieved. Participants were interviewed three times each and data material was therefore considered rich and enabled a deepened understanding. First interview texts were transcribed and read separately for each participant (three interviews each) and a first naïve understanding was formulated. Thereafter, all interviews were analysed as a whole to enable an in-depth understanding of older persons' lived experiences over time. Lindseth and Norberg (2004) stress that interpretations have different meanings and there is not only one single truth possible. The meaning in this study of older persons lived experiences is one of the conceivable meanings, found by the authors as most useful. The findings in this study present knowledge that creates a foundation for other groups of older people dependent on help, if the reader decontextualizes the interpretation into their own context [18].

## 6. Conclusions and Implications for Practice

Professional carers have to acknowledge that the life situation of older persons involves an existence of living with uncertainty over time as to how they relate to their own independence and dependence as regards themselves and others. Older persons are aware of their dependence but still want to be independent. It is suggested that one should focus on seeing older persons as interdependent in the caring encounter through a relational approach on autonomy, that it is important to help dependent older persons and to acknowledge their changing vulnerability over time. Respect for older people's right to self-determination should not just be a norm to be followed at the risk of leaving them to fend for themselves'. There is a need to pay attention to continuity and the routines essential for older persons, in order to understand their everyday lives when they are being cared for by municipal home help services. The implications for practice are a care of the old which focuses on a relational approach on autonomy beyond the traditional individualistic and a person-centered practice. It makes it easier for older people to have a professional carer they trust which acknowledges their vulnerability and that their independency and dependency changes over time. Further research is needed on how professional carers can improve older people's independent decision making in the relationship through a relational approach to autonomy focusing on interdependence. 

## Figures and Tables

**Table 1:** Examples of the structural analysis.

Meaning units	Condensed meaning units	Subtheme	Theme	Main theme
Yes, they come with me and sometimes they have to go on their own if I am not well and I have to stay home. Well sometimes I go by myself…. Well it is like you look forward to something at the beginning of the week then you go shopping. But I am not allowed to really…. I am happy with the way it is…. Well I go along with the way things are. Well I wait for Friday as if it is something special	To not be able to go out and shop for oneself and to be dependent on professional carers help imply that one's wishes will be limited. It does not feel good but it will work	Neglecting one's own need to allow for the needs of others	Adjusting one's own independence and dependence with regard to oneself and others	Living with uncertainty as to how to relate one's own independence and dependence with regard to oneself and others
If the professional carer does not have the time then she tells me. Then I have to understand that she will have to rush off somewhere else. There could be other people lying on the floor needing help to get up, so that is what comes first	To accept that the professional carer says that she does not have the time to help. There may be other caretakers who need her help urgently which she must attend to first			
Well there are some who are a little bit faster but she is rarely here but that doesn't really matter. Well they sometimes are in a hurry like that…. It is not often they take their time. It is different; all girls are different and do different things. Well you know who is who and who is better than the other but you never say anything because each of them does things their way. Well I would not do it that way, but we all are different I get the help I should have anyway…	All professional carers are different; some are more stressed than others. One does not always agree with their ideas. To accept as long as they do what they should	Withholding one's thoughts		
Usually you can tell by looking at people what they want themselves and what they would think if you get too close or like that. If you ask someone about a personal matter they will tell you, if they do not like it, of course they would. But it happens so rarely so I cannot recall, you really notice on people how open they are. Well then you avoid that of course, remembering those you can't talk to anyhow or about anything. Others I can talk to about anything as if they were one's daughter coming to visit	Some professional carer is more difficult to get contact with than others. It is important to know who want to open or distance themselves. To learn gradually wish of them, it is possible to talk to or not			

**Table 2:** Subthemes, themes, and main theme.

Sub-themes	Theme	Main theme
Relying on others to manage one's life	One's independence lies in the hands of others	Living with uncertainty as to how to relate to independence and dependence with regard to oneself and others
Deciding for oneself is beyond one's reach		
Waiting for others to come		
Deciding for oneself when to become involved	One's independence lies in one's own hands	
Managing oneself with professional carers as an extended arm		
Handing over the decisions to others		
Giving and taking in the relationship with professional carers		
Neglecting one's own needs to allow for the needs of others	Adjusting one's own independence and dependence with regard to oneself and others	
Withholding one's thoughts		
